# Risk factors for thoracic and spinal deformities following lung resection in neonates, infants, and children

**DOI:** 10.1007/s00595-016-1434-1

**Published:** 2016-10-25

**Authors:** Satoshi Makita, Kenitiro Kaneko, Yasuyuki Ono, Hiroo Uchida

**Affiliations:** Department of Pediatric Surgery, Nagoya University Graduate School of Medicine, 65 Tsurumai-cho, Showa-ku, Nagoya, 466-8560 Japan

**Keywords:** Thoracoscopic surgery, Lung resection, Congenital lung disease, Late complications, Thoracic deformities, Spinal deformities

## Abstract

**Purpose:**

We aimed to identify the risk factors for thoracic and spinal deformities following lung resection during childhood and to elucidate whether thoracoscopic surgery reduces the risk of complications after lung resection.

**Methods:**

We retrospectively examined the medical records of all pediatric patients who underwent lung resection for congenital lung disease at our institution between 1989 and 2014.

**Results:**

Seventy-four patients underwent lung resection during the study period and were followed-up. The median age of the patients at the time of surgery was 5 months (range 1 day–13 years), and 22 were neonates. Thoracotomy and thoracoscopy were performed in 25 and 49 patients, respectively. Thoracic or spinal deformities occurred in 28 of the 74 patients (37%). Univariate analyses identified thoracotomy, being a neonate (age: <1 month) at the time of surgery, and being symptomatic at the time of surgery as risk factors for these deformities. However, a multivariate analysis indicated that only thoracotomy and being a neonate were risk factors for deformities.

**Conclusions:**

Thoracoscopic surgery reduced the risk of thoracic and spinal deformities following lung resection in children. We suggest that, where possible, lung resection should be avoided until 2 or 3 months of age.

## Introduction

Previous studies have reported that 18–50% of pediatric patients that undergo thoracotomy for esophageal atresia or congenital heart disease develop thoracic or spinal deformities [1–8]. However, it remains unclear how frequently deformities occur following thoracotomy for pediatric lung diseases, because only four studies have examined this topic and the reported incidence rates range from 2 to 44% [9–12]. In recent years, minimally invasive surgery has been applied to more challenging fields, including thoracic surgery for neonates and infants [13]. However, it is unknown whether recent advances in minimally invasive pediatric surgery have helped to prevent such deformities. This study aimed to identify the risk factors for thoracic and spinal deformities following lung resection in neonates, infants, and children, and to elucidate whether thoracoscopic surgery reduces the risk of complications after lung resection.

## Patients and methods

We retrospectively examined the medical records of all pediatric patients who underwent lung resections at our institution between January 1989 and December 2014. The survey was approved by the Ethics Committee of our institution (#2015-0335). We excluded patients with neoplastic disease. Thoracoscopic lung resection was introduced at our institution in 2008. The procedure was performed via 3 or 4 ports (3–5 mm) as previously described [14]. Resected specimens were removed through a 10-mm port at an enlarged initial 5-mm port incision.

Postoperative thoracic and spinal deformities were included as ‘musculoskeletal deformity’ in the Common Terminology Criteria for Adverse Events version 4.0 and included as ‘other (specify)’ in the Japan Clinical Oncology Group postoperative complications criteria [15, 16]. Postoperative thoracic and spinal deformities were classified into pectus excavatum, pectus carinatum, scoliosis, and others in this study. Chest wall deformities were diagnosed during visual examinations at our Outpatient Department. Scoliosis was diagnosed as a Cobb angle of ≥10° on radiography according to the definition of early onset scoliosis [17, 18]. Patients who developed thoracic or spinal deformities prior to surgery were excluded from the postoperative deformity group. The following information was obtained from the patients’ medical records as candidate prognostic factors for thoracic and spinal deformities: gender, affected side, type of surgical procedure performed, age at the time of surgery, and whether the patient was symptomatic at the time of surgery.

All of the statistical analyses were performed using the EZR software package (Saitama Medical Center, Jichi Medical University, Saitama, Japan), which is a graphical user interface for the R software program (The R Formulation for Statistical Computing, version 3.0.3) [19]. Univariate analyses were carried out using Fisher’s exact test for categorical variables and the Mann–Whitney *U* test for continuous variables. The variables that exhibited statistical significance in the univariate analyses were included in the multivariate analysis, which was performed using logistic regression. *P* values of <0.05 were considered to indicate statistical significance.

## Results

Eighty patients underwent lung resections during the study period. Since the introduction of thoracoscopic procedures, thoracoscopic resection was attempted in all but one of the patients (an urgent case with severe respiratory distress). The details of six patients were unclear, because they were lost to follow-up. The remaining 74 patients were analyzed. The male-to-female ratio was 1.0:1.1, and the median age of the patients at the time of surgery was 5 months (range 1 day–13 years). There were 22 neonatal patients (<30 days of age). Lung resections were performed for the following diseases: congenital pulmonary airway malformation (*n* = 51), intralobar sequestration (*n* = 6), congenital pulmonary airway malformation in conjunction with intralobar sequestration (*n* = 6), extralobar sequestration (*n* = 5), bronchial atresia (*n* = 3), lobar emphysema (*n* = 2), and a bronchial cyst (*n* = 1). Fifty-two patients underwent thoracoscopic lung resection; three were converted to thoracotomy. Thus, thoracotomy and thoracoscopy were performed in 25 and 49 patients, respectively. The details of the surgical procedures are shown in Table 1. At the time of surgery, 15 patients exhibited symptoms, such as tachypnea and retracted breathing. Thoracotomy was performed significantly more often than thoracoscopy in symptomatic patients. Conversely, the frequencies of these procedures were similar among the neonates (Table 2). The median follow-up period was 8.3 years (range, 2–14 years) in the thoracotomy group and 4 years (range 1.0–6.8 years) in the thoracoscopy group (*P* = 0.000035, Mann–Whitney *U* test).

**Table 1:** Details of the surgical procedures

Surgical procedure	Thoracotomy (*n* = 25)	Thoracoscopy (*n* = 49)
Right lobectomy		
Upper	5	4
Middle	0	2
Lower	6	14
Bilobectomy	1	1
Total resection	1	0
Partial resection/segmentectomy	1	2
Left lobectomy		
Upper	7	5
Lower	4	14
Partial resection/segmentectomy	0	7

**Table 2:** Relationships among neonates, symptoms, surgical procedures, and frequency of deformities

	*n* (%)	Thoracotomy (*n* = 25)	Thoracoscopy (*n* = 49)	*P* value^a^
Neonates that underwent lung resection	22	10	12	0.19
Frequency of deformities	14 (64)	10 (100)*	4 (33)*	0.0017*
Symptomatic patients that underwent lung resection	15	10	5	0.0049
Frequency of deformities	11 (73)	10 (100)**	1 (20)**	0.0037**
Symptomatic neonates that underwent lung resection	13	10	3	0.00046
Frequency of deformities	11 (85)	10 (100)***	1 (33)***	0.039***

* represents incidence rates in neonates that underwent lung resection; ** represents incidence rates in symptomatic patients that underwent lung resection; *** represents incidence rates in symptomatic neonates that underwent lung resection

^a^Fisher’s exact test

Thoracic or spinal deformities were observed following lung resection in 28 of the 74 patients (37%) (Table 3). Scoliosis and pectus excavatum were the most common deformities. Among these, one patient with scoliosis was treated with a brace, and three with pectus excavatum underwent surgical correction. Deformities other than scoliosis, pectus excavatum, and pectus carinatum were seen in 4 patients (5.4%), all of whom exhibited chest asymmetry. Concurrent scoliosis and pectus excavatum occurred in three patients, and concurrent scoliosis and chest asymmetry were seen in one patient. Pectus excavatum occurred significantly earlier than the other deformities (*P* = 0.0011, Mann–Whitney *U* test). Deformities after neonatal surgery included scoliosis (*n* = 3), pectus excavatum (*n* = 5), both scoliosis and pectus excavatum (*n* = 3), chest asymmetry (*n* = 2), and pectus carinatum in (*n* = 1).

**Table 3:** Deformities that occurred following lung resections

Type of deformity	*n*	(%)	Median onset time following surgery		Thoracotomy (*n* = 25)		Thoracoscopy (*n* = 49)		*P* value^a^
			Months	(Range)	*n*	(%)	*n*	(%)	
Scoliosis	15	(20)	39	(13–115)	13	(52)	2	(4)	0.0000035
Pectus excavatum	12	(16)	7*	(1–115)	8	(32)	4	(8)	0.017
Chest asymmetry	4	(5)	35	(13–71)	2	(8)	2	(4)	0.60
Pectus carinatum	1	(1)	48	(48)	0	(0)	1	(2)	1.0
All deformities	28	(37)	23	(1–115)	19	(76)	9	(18)	0.0000022

* *P* = 0.0011, Mann–Whitney *U* test

^a^Fisher’s exact test

Univariate analysis showed that deformities were significantly more common in patients who underwent thoracotomy, patients who underwent lung resection during the neonatal period, and symptomatic patients (Table 4). No significant differences were observed in the extent of the resection. The relationships among neonatal age (<1 month), being symptomatic at the time of surgery, and/or the type of surgical procedure and the frequency of deformities are shown in Table 2. Thoracoscopic surgery caused nine deformities (Tables 3, 4). Four of these patients had undergone lung resections during the neonatal period, and two had undergone lung resection at 1 month of age. Among the patients that underwent lung resection during the neonatal period, deformities occurred significantly more often after thoracotomy than after thoracoscopy (Table 2).

**Table 4:** Prognostic factors (univariate analysis)

Variable	Deformity		*P* value
	*n*	(%)	
Gender			
M (*n* = 35)	14	(40)	0.72
F (*n* = 39)	14	(36)	
Affected side			
R (*n* = 37)	13	(35)	0.63
L (*n* = 37)	15	(41)	
Surgical procedure			
Thoracotomy (*n* = 25)	19	(76)	0.0000022
Thoracoscopy (*n* = 49)	9	(18)	
Lobectomy or partial resection (*n* = 71)	26	(37)	0.66
Bilobectomy or total resection (*n* = 3)	2	(67)	
Age at the time of surgery			
<1 month (*n* = 22)	14	(58)	0.0041
≥1 month (*n* = 52)	14	(26)	
<3 months (*n* = 31)	16	(52)	0.053
≥3 months (*n* = 43)	12	(28)	
Symptomatic at the time of surgery			
Yes (*n* = 15)	11	(73)	0.0025
No (*n* = 59)	17	(29)	

A multivariate analysis demonstrated that thoracotomy and being a neonate (age: <1 month) were independent risk factors for spinal or thoracic deformities following thoracoscopic surgery (Table 5).

**Table 5:** Prognostic factors (multivariate analysis)

Prognostic factor	Odds ratio (95% CI)	*P* value
Thoracotomy	14.10 (3.84–51.80)	0.000066
Age <1 month	4.44 (1.03–19.20)	0.045
Symptomatic	1.75 (0.30–10.20)	0.53

*CI* confidence interval

## Discussion

This study shows that thoracic and spinal deformities frequently occurred following lung resection during childhood (overall rate: 38%; post-thoracotomy rate: 76%) and that thoracotomy and undergoing lung resection during the neonatal period were independent risk factors. This study demonstrates that thoracoscopic surgery reduced the risk of complications after lung resection.

During infancy, the skeletal muscles are developing and cutting the latissimus dorsi muscle and/or the serratus anterior muscle during posterolateral thoracotomy can result in postoperative muscular atrophy, which can cause the development of thoracic and/or spinal deformities [2–4, 6, 7]. Axillary thoracotomy or other muscle-sparing thoracotomies might avoid muscular atrophy, but we did not use these procedures. Thoracoscopic lung resection can avoid this and—as shown here—contribute to a reduction in deformities. Similarly, Lawal et al. reported that thoracoscopic surgery significantly reduced the incidence of scoliosis (from 54 to 10%); however, the study included procedures other than lung resection [8]. Despite the lack of a control group, Albanese and Rothenberg reported that there were no cases of musculoskeletal deformity following 144 thoracoscopic lobectomy procedures during a follow-up period of 1–10 years [20].

Negative intrapleural pressure is another possible mechanism that may lead to the development of deformities after lung resection. This can be seen in patients with congenital diaphragmatic defects who exhibit pulmonary hypoplasia on the affected side [21]. The negative intrapleural pressure generated by upper airway obstruction in laryngomalacia has also been reported to be related to pectus deformity [22]. In this mechanism, the thorax is deformed because of the negative intrapleural pressure due to the dead space in the thoracic cavity following the resection of space-occupying lesions. The fragile neonatal thorax may be more susceptible to deformities caused by negative intrapleural pressure than the thoraces of older patients. The most dramatic changes in sternum and rib cage morphology occur in childhood when these structures are undergoing ossification [23, 24]. The degree of ossification in neonates, which is the lowest among the developmental stages, may be responsible for the neonatal period being an independent risk factor for thoracic or spinal deformities after lung resection. Based on this result, we have changed our opinion that cystic lesions should be removed during the neonatal period [14]. Thoracoscopic lobectomy for asymptomatic congenital cystic lung disease should be performed later. However, it is important that resection be performed before 6 months of age, because asymptomatic cystic lesions tend to become symptomatic after 6 months, and because inflammation is present even in asymptomatic cases [25–27].

Our patients had an unexpectedly high frequency of thoracic and spinal deformities. One possible reason is that we examined both thoracic and spinal deformities, while the previous studies examined either type of deformity alone. Another reason is that our follow-up period was longer than the follow-up periods reported in the previous studies [9–12]. The majority of deformities, with the exception of pectus excavatum, develop at 3–4 years after surgery; thus, a long follow-up period is required to evaluate the musculoskeletal anomalies that occur following lung resection. Finally, even if mild scoliosis was not diagnosed by a physical examination, the rate at which it was diagnosed on radiography was higher than expected; thus, we consider our findings to be more accurate than those reported in the previous studies.
